# Management styles in outpatient nursing work: impacts on the worker’s health^*^

**DOI:** 10.1590/1980-220X-REEUSP-2022-0127en

**Published:** 2022-11-07

**Authors:** Katerine Moraes dos Santos, Gisele Massante Peixoto Tracera, Kayo Henrique Jardel Feitosa Sousa, Allan Marcos da Silva Palheta, Rosângela Marion da Silva, Regina Célia Gollner Zeitoune

**Affiliations:** 1 Universidade Federal do Rio de Janeiro, Instituto de Atenção à Saúde São Francisco de Assis, Rio de Janeiro, RJ, Brazil.; 2 Universidade Federal do Estado do Rio de Janeiro, Hospital Universitário Gafreé & Guinle, Rio de Janeiro, RJ, Brazil.; 3 Universidade do Estado do Rio de Janeiro, Policlínica Piquet Carneiro, Rio de Janeiro, RJ, Brazil.; 4 Universidade Federal do Rio de Janeiro, Escola de Enfermagem Anna Nery, Rio de Janeiro, RJ, Brazil.; 5 Universidade Federal de Santa Maria, Departamento de Enfermagem, Santa Maria, RS, Brazil.; 6 Universidade Federal do Rio de Janeiro, Escola de Enfermagem Anna Nery, Departamento de Enfermagem de Saúde Pública, Rio de Janeiro, RJ, Brazil.

**Keywords:** Nursing, Team, Occupational Health, Health Management, Personnel Management, Ambulatory Care Facilities, Grupo de Enfermería, Salud Laboral, Gestión en Salud, Administración de Personal, Instituciones de Atención Ambulatoria, Equipe de Enfermagem, Saúde do Trabalhador, Gestão em saúde, Gestão de Recursos Humanos, Instituições de Assistência Ambulatorial

## Abstract

**Objective:**

To analyze management styles in outpatient clinics of university hospitals and their impacts on the nursing workers’ health.

**Method:**

Quantitative, cross-sectional study with 388 nursing professionals working in 11 outpatient clinics linked to public universities in Rio de Janeiro. The Management Styles Scale, the Pathogenic Suffering at Work Scale, and the Work-Related Physical and Psychosocial Harms Scale were used.

**Results:**

The managerial and collective management styles showed a moderate presence for the outpatient clinics nursing staff. The characteristics of the predominantly managerial management style, evidenced by the lack of participation in decision-making, the strongly hierarchical work, focused on norms and control, acted as predictors of the experiences of suffering and of the physical, psychological, and social damages presented by the professionals working in this context.

**Conclusion:**

The analysis of management styles allowed elucidating characteristics that have the potential to negatively impact the workers’ health, highlighting the need to review the management models currently adopted for the outpatient nursing team.

## RESUMO

**Objetivo:**

Analisar os estilos de gestão em ambulatórios de hospitais universitários e seus impactos na saúde dos trabalhadores de enfermagem.

**Método:**

Estudo quantitativo, transversal, participaram 388 profissionais de enfermagem atuantes em 11 ambulatórios vinculados a universidades públicas no Rio de Janeiro. Utilizamos a Escala de Estilos de Gestão, a Escala de Sofrimento Patogênico no Trabalho e a Escala de Danos Físicos e Psicossociais relacionados ao Trabalho.

**Resultados:**

Os estilos de gestão gerencialista e coletivo apresentaram presença moderada para a equipe de enfermagem dos ambulatórios. As características do estilo de gestão predominantemente gerencialista, evidenciadas pela falta de participação na tomada de decisão, o trabalho fortemente hierarquizado, focado nas normas e controle, atuaram como preditores das vivências de sofrimento e dos danos físicos, psíquicos e sociais apresentados pelos profissionais atuantes nesse contexto.

**Conclusão:**

A análise dos estilos de gestão permitiu elucidar características que têm potencial para impactar negativamente a saúde dos trabalhadores destacando-se a necessidade de rever os modelos de gestão atualmente adotados para a equipe de enfermagem ambulatorial.

## RESUMEN

**Objetivo:**

Analizar los estilos de gestión en ambulatorios de hospitales universitarios y sus impactos en la salud de los trabajadores de enfermería.

**Método:**

Estudio cuantitativo, transversal, participaron 388 profesionales de enfermería actuantes en 11 ambulatorios vinculados a las universidades públicas en Rio de Janeiro. Se utilizó la Escala de Estilos de Gestión, la Escala de Sufrimiento Patogénico en el Trabajo y la Escala de Daños Físicos y Psicosociales relacionados al Trabajo.

**Resultados:**

Los estilos de gestión gerencial y colectiva presentaron presencia moderada en el equipo de enfermería de los ambulatorios. Las características del estilo de gestión predominantemente gerencial, evidenciadas por la falta de participación en la toma de decisión, el trabajo fuertemente jerarquizado, enfocado en las normas actuaron como predictores de las vivencias de sufrimiento y de los daños físicos, psíquicos y sociales presentados por los profesionales actuantes en ese contexto.

**Conclusión:**

El análisis de los estilos de gestión permitió elucidar características que tienen potencial para impactar negativamente la salud de los trabajadores destacándose la necesidad de revisar los modelos de gestión actualmente adoptados para el equipo de enfermería ambulatorial.

## INTRODUCTION

Public organizations are complex systems, in which the conditions and organization of work are influenced by the organizational culture, permeated by normative aspects, characterized by a bureaucratic space, with party political interference, centralization of decision-making with little scope remaining for the action of local administration and project interruption due to scarcity of resources^(1)^.

The forms of management and organization of work have repercussions on the public health sector and, more specifically, on nursing work, and are perceived by professionals in their daily work. The different management forms and models have the main objective of instituting the best practices in organizations, impacting the work and the quality of life at work^(2)^.

The health professionals’ work context is permeated by difficulties, frustrations, and suffering, being far from the interests, needs, and aspirations of the workers and users, who often have their work made difficult and even impossible due to the lack of material conditions necessary to achieve what is demanded from them^(3)^.

University hospitals (*HUs*) – inserted in this scenario – have unquestionable social importance in Brazil. They are publicly funded institutions that serve the entire population and are a reference for medium and high complexity services in the Brazilian Public Health System (*SUS*). Despite this importance, there are challenges concerning the organization and management of the work of these hospitals, protection and production of workers’ health, which are related to the deficit of employees, inadequacy of physical space, insufficiency and precariousness of materials, and lack of beds. Such aspects hinder the compliance with biosafety standards, overload professionals, and alert to the exposure of these workers to psychosocial risks^(4)^.

Nursing work is developed in this context and is permeated by management activities, which can contribute to the workers’ health, or contribute to their illness. This study is based on the concept that it is important to evaluate organizational management modes, given that these are predictors of experiences of suffering and psychological and social damage, impacting the worker’s health^(5)^.

Therefore, the Protocol for the Assessment of Psychosocial Risks at Work (PROART) was used as framework^(5)^. This conceptual model considers that two management styles can coexist in organizations: the Managerial Style and the Collective Style. The Managerial Style “is characterized by the centralization of decisions in the role of the manager, strong bureaucratic system, valuing of rules to the detriment of subjects, rigid disciplinary system, and high control of work”^(5:239)^; and the Collective Style, on the other hand, “is characterized by well-established exchange relationships among members, valuing of creativity and innovation, furtherance of professional interaction and promotion of better people’s well-being, with flexibility in the hierarchical levels, and valuing of recognition and commitment to work”^(5:240)^.

Based on the practical experience in the daily life of nursing in different contexts, it is observed that there are aspects inherent to the two management styles permeating the occupation. Therefore, organizations where the Managerial Style predominates point to psychosocial risks and can negatively impact the workers’ health, while the Collective Style has positive characteristics from the pespective of the worker’s health^(5)^.

It should be noted, however, that outpatient clinics differ from other sectors of nursing practice, in which there are no shifts, the patient being treated is usually stable and the appointments are scheduled and show regular demand. Considering the gap in scientific production related to the nursing practice in outpatient settings^(6)^ and that investigations focusing on management styles and their influence on the nursing workers’ suffering and illness processes were not observed, this research aimed to collaborate in this discussion.

Thus, this study aimed at analyzing management styles in outpatient clinics of university hospitals and their impacts on the nursing workers’ health.

## METHOD

### Design of Study

Quantitative study with a cross-sectional approach.

### Population

Nursing team workers.

### Local

The research was carried out in 11 outpatient clinics linked to university hospitals and specialized outpatient units of three public universities located in the city of Rio de Janeiro, RJ, Brazil.

### Selection Criteria

The inclusion criterion was to be a nurse, nursing technician or assistant working in nursing care, and the exclusion criterion was being a professional on extended leaves. The total study population accounted for 604 professionals at the time, of which 483 met the inclusion criteria; 121 professionals who did not perform activities in nursing care were excluded. There were 95 losses, these being related to the professionals who were approached, but did not return the instruments, and in the end, 388 professionals remained.

### Sample Definition

Initially, the proposal was to carry out a census, so that all eligible professionals who were found during the data collection stage were invited to participate in the research. However, a period of two months was established for the data collection stage in each of the 11 units, in view of the possibility of simultaneous collection, with a total of six months dedicated to this stage.

Thus, a calculation was performed using the software OpenEpi^(7)^ for estimating the sample size, to significantly represent the population surveyed. The total of 388 professionals represented a confidence interval of 99.9%, with this being representative of the population studied.

### Data Collection

Data collection took place from July to December 2018, during the opening hours of the clinics, from Monday to Friday, from 7 am to 7 pm. It was carried out by the principal investigator and a team of research assistants, one being an undergraduate nursing student and two students from a graduate nursing program. The team was previously trained to apply the instruments. The workers were individually invited to participate in the study, and upon acceptance, the Free and Informed Consent Form was delivered, its reading and subsequent signature requested, with the conditions exposed. Then, the data collection instruments were delivered in a non-transparent envelope, and a date for return was scheduled.

A questionnaire for sociodemographic and occupational characterization, the Management Styles Scale (EEG), the Pathogenic Suffering at Work Scale (ESPT), and the Work-related Physical and Psychosocial Harms Scale (EDT), which are part of the Protocol of Assessment of Work-Related Psychosocial Risks (PROART), were used. PROART is validated for use with workers and its instruments are Likert-type frequency scales, consisting of five points: 1 (never), 2 (rarely), 3 (sometimes), 4 (often), and 5 (always)^(5,8)^.

The EEG, made up of 22 items, allows to apprehend the ways of feeling, thinking, and acting shared in the organization, constituting the adopted management style and being composed of two factors: Managerial Style (10 items) and Collective Style (12 items)^(5)^. According to the authors, the predominance of characteristics of the managerial style points to negative results for the workers’ health; in contrast, the predominance of characteristics of the collective style is positive.

The ESPT, consisting of 27 items and formed by three factors: Lack of meaning at work (nine items), which is expressed by feelings of worthlessness when doing work that makes no sense to oneself and is not important or meaningful to the organization, customers, and society; Mental exhaustion (eight items), which is characterized by feelings of injustice, discouragement, dissatisfaction, and weariness with work; and Lack of recognition (10 items), which translates into feelings of devaluation, non-acceptance, and/or non-admiration by colleagues and supervisors, and lack of freedom to express what he/she thinks and feels in relation to work^(5)^.

The EDT has 23 items, subdivided into three factors that refer to the psychological, social, and physical dysfunctions generated by the confrontation with the organization of work and their respective management styles and experiences of suffering. Physical damage (nine items) is related to body pain and biological disorders; psychological damage (seven items) is defined as negative feelings about oneself and life in general; and social damage (seven items) is characterized by isolation and difficulties in family and social relationships^(5,8)^.

### Data Analysis and Treatment

Data were organized, processed, and analyzed using the software *Statistical Package for the Social Sciences*, version 21.0.

The interpretation of the EEG, ESPT, and EDT scales was performed based on the analysis of the general mean and standard deviation of the factor, which was carried out from the mean and standard deviation of each item composing the factor, later using the aid of the SPSS software and items were grouped to form factors. For the EEG, the analysis of the three factor items evaluated with higher means was also performed to check which situations influenced the general results.

The EEG has 3.00 as a mean point; its classification considers the proximity of the item results to that point, with values above 3.50 being classified as the predominant presence of the managerial style; moderate, when between 2.50 and 3.49; and, uncharacteristic of the management style when values were lower than 2.50^(5,8)^.

In ESPT and EDT, the items are negative, that is, the higher the score, the greater the psychosocial risks. Considering the standard deviation in relation to the mean point, the parameters for evaluating the mean, standard deviation and frequency of the factor are as follows: 1.00 to 2.29, low psychosocial risk, which represents a positive result; 2.30 to 3.69, medium risk, which represents a state of alert/limit situation for psychosocial risks at work, demanding short- and medium-term interventions; 3.70 to 5.00, high psychosocial risk, negative result, demands immediate interventions in the causes, aiming to eliminate and/or mitigate them^(5,8)^.

For the bivariate statistical analysis, considering that the low risk classification is the only one that indicates a positive result for the workers’ health, the medium and high risks were added and considered as a dependent variable for the analysis of the ESPT and EDT scales. For the EEG, the categories moderate and predominant presence were added, and it was considered an independent variable. This way, bivariate analyses were performed using the odds ratio (OR), with a confidence interval of 95%, with a significance level of 5% to verify the association between managerial and collective management styles and medium or high psychosocial risk of pathogenic suffering and physical, psychological, and social damage for the population studied.

For this analysis, the association among the scales was performed as established by Facas^(8)^, considering that the management styles adopted are predictors of workers’ experiences of suffering and physical and psychosocial damage.

### Ethical Aspects

The study complied with all ethical precepts for research involving human beings, as recommended by Resolution No. 466/2012, being approved in 2018 by the Research Ethics Committee of the proposing institution and by seven co-participants: 1. CEP-EEAN/HESFA/UFRJ, opinion No. 2.567.807; 2. CEP-IPUB/UFRJ, opinion No. 2.617.252; 3. CEP-HUCFF/IDT/IG/UFRJ, opinion No. 2.663.714; 4. CEP-ME/UFRJ, opinion No. 2.621.377; 5. CEP-IPPMG/UFRJ, opinion No. 2.743.032; 6. CEP-INDC/UFRJ, opinion No. 2.870.894; 7. CEP-HUGG/UNIRIO, opinion No. 2.617.172; 8. CEP-HUPE/PPC/UERJ, opinion No. 2.612.075. All participants signed the Free and Informed Consent Form.

## RESULTS

Among the nursing team professionals who participated in the research, those who declared themselves to be female predominated (88.6%, n = 344), with a mean age of 48 years (SD ± 11), married or in a common-law relationship (52.6%, n = 204), with children (69.8%, n = 271), of non-white race/color (61.6%, n = 239), with higher education (68.3%, n = 265), nursing technicians or assistants (69.6%, n = 270), with a permanent contract (90.7%, n = 352), only one employment contract (52.1%, n = 202), working hours of more than 30 hours per week (61.6%, n = 239), median of 25 years of work in nursing, 18 years in the institution, and 5.5 years in the outpatient’s clinic.

In the analysis of the mean and standard deviation of the factor, it was observed that the two styles had a moderate presence for the nursing staff of the university outpatient clinics, with the Managerial Management Style with μ = 2.66, SD = 0.7, and the Collective Management Style with μ = 2.99, SD = 0.9. The standard deviation below one also stands out, which means that the mean was representative of the whole.

Table 1 presents the items of the Managerial and Collective Management Style factors, in descending order, starting from the average of each item, allowing the observation of those presenting the highest averages of each factor.

**Table 1.** Items of the factors of the Management Styles Scale according to mean and standard deviation for nursing workers from outpatient clinics of university hospitals in the city of Rio de Janeiro – Rio de Janeiro, RJ, Brazil, 2018. (n = 388)

EEG management style factors items	Mean	SD	Evaluation
**Managerial Style**	**2.66**	**0.7**	**Moderate**
Hierarchy is valued in this organization	3.69	1.1	Predominant
Great importance is credited to the rules in this organization	3.27	1.1	Moderate
There is strong control of the work	3.09	1.1	Moderate
The work environment becomes disorganized with changes	2.78	1.2	Moderate
Affective ties among people in this organization are weak	2.72	1.2	Moderate
Here managers prefer to work individually	2.44	1.3	Uncharacteristic
In this organization, managers consider themselves the center of the world	2.31	1.4	Uncharacteristic
The managers of this organization consider themselves irreplaceable	2.30	1.3	Uncharacteristic
The managers of this organization will do anything to get attention	2.11	1.2	Uncharacteristic
In my work, idolatry of bosses is encouraged	1.86	1.2	Uncharacteristic
**Collective Style**	**2.99**	**0.9**	**Moderate**
People are committed to the organization even when there is no adequate return	3.66	1.1	Predominant
Collective work is valued by managers	3.35	1.2	Moderate
For this organization, the result of the work is seen as an achievement of the group	3.29	1.2	Moderate
The merit of the achievements in the company belongs to everyone	3.14	1.3	Moderate
The competence of the workers is valued by the management	3.01	1.2	Moderate
Innovation is valued in this organization	2.95	1.2	Moderate
Managers care about the workers’ well-being	2.94	1.2	Moderate
Managers favor the interactive work of professionals from different areas	2.82	1.3	Moderate
There is rigorous planning of actions	2.78	1.1	Moderate
We are encouraged by managers to seek new challenges	2.73	1.2	Moderate
Decisions in this organization are made in a group	2.70	1.2	Moderate
There are similar ascension opportunities for all people	2.53	1.3	Moderate

Table 2 shows the association of managerial and collective management styles with medium or high risk assessed by nursing professionals for the factors lack of meaning at work, mental exhaustion, and lack of recognition of the Pathogenic Suffering Scale at Work, and physical, psychological, and social damage from the Work-Related Injury Scale.

**Table 2.** Association between the factors of the Management Styles Scale (EEG) and the factors of the Pathogenic Suffering at Work Scale (PTS) and the Physical and Psychosocial Harm at Work (EDT) Scale. Analyses performed based on the odds ratio (OR) and respective confidence interval (95%CI) – Rio de Janeiro, RJ, Brazil, 2018.

Management Styles Scale (EEG)	Pathogenic Suffering at Work Scale (ESPT)						Scale of Physical and Psychosocial Harm at Work (EDT)					
	Lack of meaning at work		Mental exhaustion		Lack of recognition		Psychological Damage		Social Damage		Physical Damage	
	Mean/high risk n(%) alto n(%)	OR (95%CI)	Mean / high risk n(%)	OR (95%CI)	Mean / high risk n(%)	OR (95%CI)	Mean/high risk/ n(%)	OR (95%CI)	Mean/high risk n(%)	OR (95%CI)	Mean/high risk n(%)	OR (95%CI)
**Managerial**												
Uncharacteristic	6 (3.2)	1.0	71 (38.2)	1.0	10 (5.3)	1.0	14 (7.5)	1.0	18 (9.6)	1.0	97 (51.9)	1.0
Moderate/predominant	39 (19.5)	**7.30** **(3.01–17.71)**	131 (65.5)	**3.07** **(2.03–4.65)**	67 (33.5)	**8.91** **(4.42–17.98)**	60 (30.3)	**5.29** **(2.84–9.87)**	49 (24.6)	**3.06** **(1.71–5.49)**	152 (76)	**2.94** **(1.90–4.53)**
**Collective**												
Uncharacteristic	31 (22.8)	1.0	96 (71.1)	1.0	55 (40.4)	1.0	45 (33.1)	1.0	35 (25.9)	1.0	103 (75.7)	1.0
Moderate/predominant	14 (5.6)	**0.20** **(0.10–0.39)**	107 (42.8)	**0.30** **(0.19–0.47)**	23 (9.2)	**0.15** **(0.09–0.26)**	30 (12.0)	**0.27** **(016–0.46)**	33 (13.2)	**0.43** **(0.25–0.74)**	147 (58.8)	**0.46** **(0.29–0.73)**

## DISCUSSION

For the nursing workers of the investigated outpatient clinics, the two management styles were evaluated as having a moderate presence. An item of the managerial management style was evaluated as a predominant pattern and concerns the valorization of hierarchy in the organization. Among the items evaluated as moderate presence, the importance of rules and strong control of work stood out with the highest averages. Similarly, in the collective style, one item was evaluated as the predominant pattern and reflected people’s commitment to the organization even without adequate feedback. The other items were evaluated as moderate presence, with the highest averages highlighting the appreciation of collective work by managers and the result of the work seen as an achievement of the group.

The Management Styles Scale is a predictor of suffering and damage at work, being an inversely proportional relationship, that is, an organization that has management styles based on collective behavior, group union, and appreciation of competence and innovation favors the mobilization subjectivity of practical intelligence, inspiring collective spaces for discussion and recognition of workers. On the other hand, a rigid work organization and more individualistic and normative management styles hinder re-signification of suffering and lead to the pathogenic fate of this work and, as a consequence, to damages^(8)^.

From this perspective, the analysis of the association between management style (EGG) and pathogenic suffering at work (ESPT) showed that when there is a predominance of managerial management, there is an increased chance of the presence of the feeling of lack of meaning at work (OR = 7.3), mental exhaustion (OR = 3.07), and lack of recognition (OR = 8.91) among professionals. This same predominant management style corroborated the increased chance of physical (OR = 2.94), psychological (OR = 5.29), and social (OR = 3.06) damage among outpatient nursing workers, in the analysis of the association between management style (EGG) and physical and psychosocial harm at work (EDT).

Based on this analysis, it is worth noting that the exposure of nursing workers to work environments in which characteristics of this management style predominate have increased the chance of pathogenic suffering and work-related damage, with emphasis on the lack of recognition and psychological damage. At the same time, the characteristics of the collective management style, evidenced in the outpatient clinics by the appreciation of collective work and commitment of people, characteristics that allow for professional recognition and interaction, acted as protectors for pathogenic suffering and damage at work,when compared to the managerial management style.

The perception of aspects related to the valorization of hierarchy, rules and control over work is related to the very organization of nursing work, in which there will always be a relationship of subordination of assistants and technicians to the nurse, who, according to the nursing category legislation, is responsible for the team management and leadership.

In the technical division of work in nursing, technicians and assistants are pressured by nurses to carry out activities and tasks, but they are also demanded by other members of the health team and by users. This hierarchical organization impacts on the intensification of work that is developed in an already precarious context^(9)^, bringing consequences to these workers’ health.

In addition to the relationships established within the team itself, it is worth considering that health work is developed based on the biomedical model, which reduces the potency of interdisciplinary work, causing frustration in workers^(10)^ and impacting the autonomy of the nursing professional. In this regard, organizational and management models compromise the autonomy of this professional, who executes, but has little participation in the decision-making process, especially in work settings with hierarchical bureaucratic structures and inflexible attitudes on the part of the institution^(11)^.

Direct administration, in public health services, denotes great difficulties in managing health services, related to “low operational capacity associated with lack of budgetary, financial and administrative autonomy, low quality controls, foreign political influence, excessive bureaucracy to hire personnel, make structural adjustments and buy supplies, medicines and equipment”^(12:7)^.

To avoid this scenario, “it is necessary to invest in leadership models that allow collective and assertive decision-making, including workers in the reflective process through intense and dialogic communication, transposing evidence into practice and practice into evidence”^(13:1)^. Such models are in line with the collective management style, in which the valorization of workers’ competence and innovation, interactive work, the union of groups and shared decision-making predominate.

In the present research, the characteristics of the collective management style positively interfered with the health of nursing professionals, where this proved to be a protective factor for the feeling of lack of meaning at work (OR = 0.20), mental exhaustion (OR = 0.30) and lack of recognition (OR = 0.15) in association with pathogenic suffering at work (PTS), as well as the association with physical and psychosocial harm at work (EDT) with regard to physical (OR = 0.27), psychological (OR = 0.43), and social (OR = 0.46) harm among nursing workers. These characteristics lead to collaborative and cooperative work and to good socio-professional relationships between boss and subordinates, and also between users and professionals.

In this regard, longitudinal research carried out with nursing professionals in Sweden identified that receiving little or no support and encouragement from superiors and co-workers was related to an increased risk of disability retirement. For the researchers, the association between low social support and disability retirement can be explained by the fact that it is a professional activity developed in a team. Therefore, when the team is not supportive, it contributes to strong psychosocial tensions. In addition, as a profession involved in complex care that requires difficult decision-making, having supervisor feedback is especially important. In this sense, they conclude that social support can be a crucial factor for a healthy and stable workforce in the health area^(14)^.

On the other hand, a systematic review found that problems in the relationship of the teams can influence the worsening of the quality of care provided^(15)^. In this logic, work organization is the result of social relations, and “if real work works well, consensus is produced in the work group”, which become “new work rules”. The cohesion of these groups comes from the mutual trust, favoring cooperation and demonstrating that workers are aware of the rules, which may not be followed so that they can deal with the unforeseen, “faced by the workers’ intelligence, creativity, and ingenuity”^(16:07)^.

In this research, the socio-professional relationships established may be influencing the cohesion of this collective of workers and allowing the construction of consensus to face the real work of nursing, which is always full of unforeseen events, allowing the expression of the singularity of the subjects and the use of their creativity, mitigating the negative aspects identified.

Based on this line of interpretation, research carried out with professionals working in primary care units in violent territories identified that socio-professional relationships acted as defensive strategies, mitigating the daily suffering faced by workers, in addition to providing them with a group identity, strengthening relationships and allowing coping with tensions caused by armed violence in the territory^(17)^. This shows the importance of socio-professional relationships as mediators of suffering at work.

Regarding this aspect, a study carried out with nurses in hospitals in China identified a higher probability of permanence in their current positions and lower intention to leave work among those happy with the respect they received from patients, who had social recognition and a relationship of trust with the medical team^(18)^.

The importance of social support is revealed as a coping resource for workers^(19)^. Therefore, actions aimed at managing psychosocial risks at work must be based on measures that consider organizational aspects beyond individual issues^(20)^. A study carried out in Portugal on priority areas in the safety of nursing professionals revealed that among the twelve areas identified as priorities in occupational safety are ergonomics, communication, burnout, work breaks, and the work structure^(21)^.

From this perspective, a satisfactory work experience is one related to a positive work environment, permeated by ethics, good teamwork relationships and support, as well as opportunities for continuing education and career development^(22)^.

Based on the data presented, it appears that university outpatient clinics are work contexts in which good socio-professional relationships prevail, also revealing the importance of teamwork, an approach that derives from the management model adopted. This aspect can act in favor of the worker’s health, as there is an appreciation of collective work and the group is committed to the work performed, consequently favoring recognition mechanisms.

On the other hand, regarding the negative aspects identified, related to the characteristics of the managerial management style, it was observed that they have increased the chance of unfavorable outcomes for the nursing team, leading to pathogenic suffering and, as a consequence, to damage at work. Such aspects must be considered within organizations aimed at rethinking the management styles adopted for nursing teams, through the impact on the health of these workers, who are essential in the health work process.

This study has as a limitation its cross-sectional design, which does not allow infering temporality and directionality between the dependent and independent variables, only the associations between them; a second aspect that shall be highlighted in all research in the area of workers’ health is the “healthy worker effect”, as only professionals who were working at the time of data collection participated in the research, and it was not possible to investigate those who may have been away from work due to the consequences of work-related psychosocial risks, which may underestimate some results. It is worth noting that data collection was carried out in 2018 and the results may no longer reflect the reality of the services.

Another limitation refers to the outpatient model adopted in Brazil, which differs from other countries, hindering the comparison of results with other realities. In addition, there are few studies in Brazil focusing on outpatient units.

## CONCLUSION

As presented in this research, the psychosocial risks related to management styles have impacted the suffering and illness of these workers, but there are positive aspects that may be mitigating this outcome. The data presented evidence the presence of two management styles in university outpatient clinics, with the positive aspects being the commitment of the nursing team with the organization and the valorization of collective and collaborative work, prioritizing well-being and professional interaction, with positive socio-professional relationships. Despite this, the lack of participation in decision-making and the characteristic of highly hierarchical work, focused on norms and control, were presented as negative aspects. This has been widely corroborated by the literature.

The analysis of management styles allowed elucidating characteristics that have the potential to negatively impact the workers’ health, with the influence of managerial management being highlighted in the pathogenic suffering and illness of nursing workers. Based on the above, the need to review the management models currently adopted for the outpatient nursing team is highlighted. In this regard, we can see the importance of adopting strategies that protect the organization, its workers and its users, through practices that favor recognition, effective and respectful communication, decision-making in a shared way, allowing more autonomy to the nursing, favoring this group of workers’ health.
